# Apolipoprotein E and Atherosclerosis: From Lipoprotein Metabolism to MicroRNA Control of Inflammation

**DOI:** 10.3390/jcdd5020030

**Published:** 2018-05-23

**Authors:** Laura Bouchareychas, Robert L. Raffai

**Affiliations:** Department of Surgery, University of California San Francisco & Veterans Affairs Medical Center, 4150 Clement Street, San Francisco, CA 94121, USA; laura.bouchareychas@ucsf.edu

**Keywords:** apolipoprotein E, hyperlipidemia, atherosclerosis, inflammation, microRNA

## Abstract

Apolipoprotein (apo) E stands out among plasma apolipoproteins through its unprecedented ability to protect against atherosclerosis. Although best recognized for its ability to mediate plasma lipoprotein clearance in the liver and protect against macrophage foam cell formation, our recent understanding of the influence that apoE can exert to control atherosclerosis has significantly widened. Among apoE’s newfound athero-protective properties include an ability to control exaggerated hematopoiesis, blood monocyte activation and aortic stiffening in mice with hyperlipidemia. Mechanisms responsible for these exciting new properties extend beyond apoE’s ability to prevent cellular lipid excess. Rather, new findings have revealed a role for apoE in regulating microRNA-controlled cellular signaling in cells of the immune system and vascular wall. Remarkably, infusions of apoE-responsive microRNA mimics were shown to substitute for apoE in protecting against systemic and vascular inflammation to suppress atherosclerosis in mice with hyperlipidemia. Finally, more recent evidence suggests that apoE may control the release of microvesicles that could modulate cellular signaling, inflammation and atherosclerosis at a distance. These exciting new findings position apoE within the emerging field of intercellular communication that could introduce new approaches to control atherosclerosis cardiovascular disease.

## 1. Introduction

Apolipoprotein (apo) E is recognized for its unparalleled ability to suppress atherosclerosis [1,2,3]. Beyond its participation in the removal of atherogenic remnant lipoproteins from plasma, apoE is known to exert an influence on numerous cells including those of the vessel wall, the immune system and the bone marrow. The expression of apoE in the macrophage has long been recognized to suppress atherosclerosis by preventing foam cell formation in the vessel wall [4,5]. More recently, apoE has been shown to suppress myelopoeisis [6] and the expansion and activation of monocytes in the circulation of hyperlipidemic mice [7]. The underlying mechanism for this new protective property has largely been ascribed to the lipid efflux capacity of apoE both through its cellular expression [6] and by its ability to enhance the lipid efflux capacity of plasma HDL [7]. Beyond its properties to maintain lipid homeostasis in the circulation and immune cells, apoE is also known to control cellular signaling via its interaction with apoE receptors and heparin sulfate proteoglycans (HSPG). Through such cell-signaling properties, apoE has been shown to control biological effects ranging from macrophage plasticity to smooth muscle cell proliferation and endothelial cell activation [5]. An exciting new cell-signaling property of apoE introduces a novel paradigm for inflammation control in the cardiovascular system. Lipoprotein-associated apoE was found to suppress smooth muscle cell proliferation and aortic stiffening by regulating the expression of microRNA including by reducing levels of miR-221/222 and increasing levels of miR-145 respectively [8,9]. Furthermore, apoE was found to influence levels of cellular miR-146a in monocytes and macrophages to suppress NF-κB–driven inflammation and atherosclerosis in hyperlipidemic mice [10]. Collectively, such recent findings broaden our understanding and appreciation for apoE in its capacity to suppress atherosclerosis beyond modulating plasma and cellular lipid levels. This review article summarizes many of the well-accepted and more recently described atheroprotective properties of apoE. It also serves to introduce tantalizing new evidence linking apoE to extracellular RNA transport and intercellular signaling that could offer new therapeutic approaches for this rampant cardiovascular disease.

## 2. ApoE and Plasma Lipid Homeostasis

**Figure 1:**
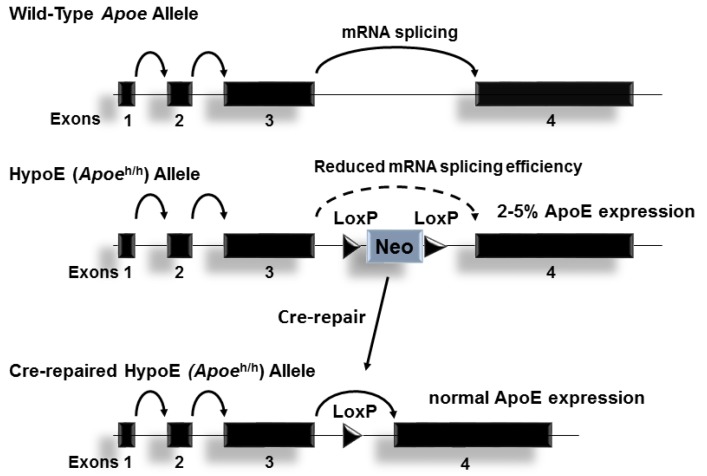
The Hypomorphic Apoe^h/h^ allele: The wildtype mouse Apoe allele is composed of 4 exons on chromosome 7. A neomycin resistance cassette flanked by loxP sites introduced into intron 3 reduces ApoE expression levels of the gene in all tissues, presumably by interrupting RNA splicing. Cre-mediated removal of neo cassette restores Apoe expression to physiological levels.

Decades of research conducted by Robert W. Mahley and Karl H. Weisgraber, along with their co-workers at the J. David Gladstone Foundation laboratories in San Francisco, California, led to seminal findings in the biology of apoE [11]. Specifically, their studies revealed structural and functional components of apoE that contribute to its ability to promote the clearance of cholesterol-rich plasma lipoproteins by the liver [11,12,13]. The “secretion-capture” process of apoE was identified as a fundamental property of hepatocyte-derived apoE that enables the protein to efficiently clear atherogenic plasma lipoproteins within the Space of Disse in the liver [14]. Although extra-hepatic sources of apoE have been shown to contribute to lipoprotein clearance by the liver [15], studies conducted with the hypomorphic apoE (*Apoe*^h/h^) mouse model of conditional apoE expression, also termed the HypoE mouse [16], demonstrated that even a very small amount of hepatocyte-derived apoE is highly effective in clearing atherogenic lipoproteins from plasma [17]. The unique phenotype of inducible apoE expression displayed by HypoE mice (Figure 1) offered new opportunities to investigate the importance in the source of apoE in the process of atherosclerosis. Furthermore, temporal Cre-mediated gene repair of the *Apoe*^h/h^ locus revealed a role for apoE in promoting the regression of atherosclerosis beyond reducing plasma lipid levels [18]. Breeding *Apoe*^h/h^ mice with mice deficient in LDL receptor gene expression led to new opportunities to study the process of apoE in atherosclerosis progression [7] and its regression [19]. Likewise, breeding *Apoe*^h/h^ mice with mice deficient in the scavenger receptor class B type-1 (SRB1^−/−^*Apoe*^h/h^ mice) led to a model of diet-induced occlusive coronary atherosclerosis, myocardial infarction and fatal ischemic heart disease [20,21]. Remarkably, temporal repair of the HypoE allele in SRB1^−/−^*Apoe*^h/h^Mx1-Cre mice led to a model of chronic heart failure in mice that survive episodes of diet-induced myocardial infarction [22]. More recently, breeding SRB1^−/−^*Apoe*^h/h^ mice with the K14-RacV12^−/+^ mouse model of human psoriasis led to studies of chronic skin inflammation in accelerated diet-induced atherosclerosis [23]. Conditional *Apoe* gene expression in HypoE mice and its derivatives thus represents a valuable tool to model the impact of hyperlipidemia in atherosclerosis cardiovascular disease and to study the contribution of apoE in these disorders.

## 3. ApoE4 Domain Interaction and Atherosclerosis

A caveat to all studies of the HypoE mouse model detailed above rests with their expression of an apoE4-like form of mouse apoE called Arg-61 apoE, engineered to reproduce a unique biophysical property of apoE4 called domain interaction [24]. Prior studies have shown that Arg-61 apoE reproduces the VLDL preference of apoE4 while retaining high affinity to the LDLR [24]. Studies by Eberlé et al. [25] demonstrated that apoE4 domain interaction contributes to accelerate diet-induced atherosclerosis in HypoE mice expressing the Arg-61 (*Apoe*R61^h/h^) alleles. Domain interaction raised plasma apoB-lipoprotein levels and reduced apoE secretion in peritoneal macrophages while enhancing their cellular activation, including by raising cell surface MHC-II expression [25]. Thus, phenotypes of inflammation and atherosclerosis reported in studies making use of HypoE mice and its derivatives could have been influenced through the expression of the Arg-61 apoE allele. Such effects can be further explored through studies of HypoE mice expressing wildtype mouse *Apoe*T61^h/h^ alleles that do not display apoE4 Domain Interaction and therefore are a model for human apoE3 [24,25].

## 4. Pleiotropic Properties of ApoE in Inflammation and Atherosclerosis Control

Beyond its pivotal role in regulating lipoprotein cholesterol transport, apoE is recognized for its capacity to suppress atherosclerosis by exerting multiple effects on almost every cell type found in the arterial wall [1,5,26]. Although macrophages are recognized as the main source of apoE in atheroma [4,27,28], newer findings have reported apoE expression by monocytes as a contributing source of hyperlipidemia and atherosclerosis regulation [10,29]. Cellular cholesterol accumulation in macrophages and their monocyte precursors leads to oxysterol-mediated up-regulation of liver X receptor (LXR) target genes that include ApoE, ABCA1 and ABCG1 that participate in eliminating pools of cellular cholesterol and increasing a local pool of extracellular apoE in the artery [29,30,31,32]. Sustained expression of apoE in the vessel wall in a murine model of macrophage death suppression established the importance of this source of apoE in atherosclerosis control [29]. ApoE is also known to reduce lipid oxidation [33], the activation of endothelial cells [34] and platelets [35], the phagocytotic clearance of apoptotic bodies [36], and suppresses the migration and proliferation of vascular smooth muscle cells [37,38]. Remarkably, these protective effects persist even when apoE is present at sub-physiological levels that lead to hyperlipidemia [27,39,40,41]. Studies have also shown that apoE can suppress mitogen-activated proliferation of CD4 and CD8 T cells [42,43,44], and antigen-dependent T cell activation by reducing the density of major histocompatibility (MHC) class II molecules and co-stimulatory molecules on macrophages [45]. ApoE is also known to regulate innate immunity [46] and the susceptibility to bacterial infections [47,48] and sepsis [49,50]. Interestingly, apoE expression by macrophages is itself subject to regulation by inflammatory cytokines. While TNFα increases apoE expression [51], IFNγ reduces apoE expression [52]. Thus, the diversity of cytokines generated within the arterial wall may impact the protective potential of apoE on atherosclerosis development.

## 5. ApoE Regulation of Macrophage Polarity and Inflammatory Phenotypes

Macrophages are plastic cells that exist as heterogeneous populations in atherosclerotic lesions [53,54]. Their polarity is driven through environmental cues including plasma lipid levels, cytokines, and oxidized lipids [55]. Pro-inflammatory M1 and the more recently described Mox macrophages contribute to plaque growth and instability [56,57], while alternatively activated M2 and Mres macrophages have been shown to participate in the regression of atherosclerosis [58,59] and resolution of inflammation [60]. Through interactions with the cell surface receptors VLDLR or ApoER2, apoE has been shown to reduce the expression levels of M1 macrophage markers including iNOS while increasing the expression of M2 markers including arginase-1 [61]. Through such signaling, apoE reduces the macrophages sensitivity to IFNγ, suppressing their secretion of pro-inflammatory cytokines and enhances their propensity for migration. In contrast, such signaling via apoE enhances the M2-like properties of macrophages including phagocytosis and the secretion of anti-inflammatory cytokines [61] that are recognized in the control of atherosclerosis progression and its regression [62].

## 6. Cell-Derived ApoE and Control of Lipid-Induced HSPC Proliferation

**Figure 2:**
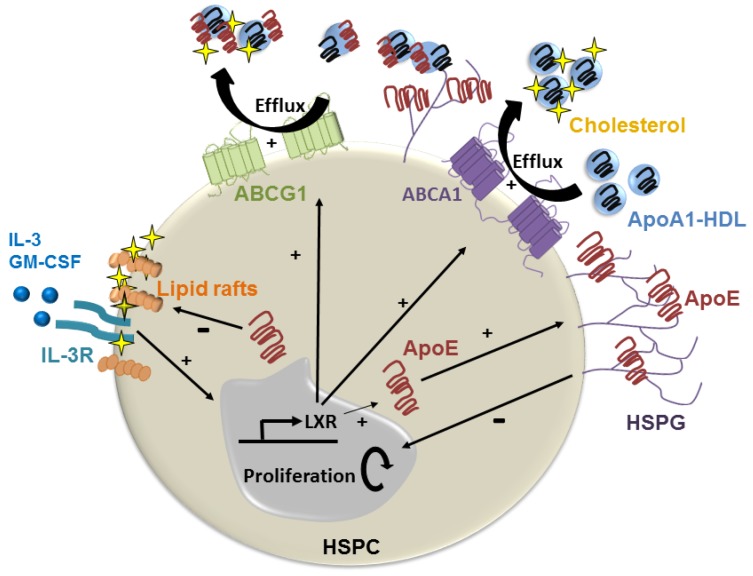
ApoE suppression of lipid-induced HSPC hyperproliferation. Hyperlipidemia promotes HSPC proliferation by enriching the plasma membrane with cholesterol-rich lipid rafts and the IL-3/GM-CSF receptor to drive growth signaling. ApoE produced by HSPC can become anchored to the cell membrane via HSPG where it promotes cholesterol efflux via HDL containing apoA1 via ABCA-1 and ABCG-1. This reduces cell surface lipid rafts, and thereby growth signaling. HDL containing apoE could also suppress HSPC proliferation by promoting cholesterol efflux via ABCG-1.

Studies by Yvan-Charvet et al. revealed that impaired cellular cholesterol efflux results in the hyperproliferation of hematopoietic stem and multipotential progenitor cells (HSPCs) in the bone marrow, causing myeloproliferation and monocytosis [63]. HSPC hyperproliferation in hyperlipidemic mice was linked to increased cholesterol-rich lipid rafts that enhanced the density of cytokine receptors in the plasma membrane and proliferative signaling [63]. Interestingly, apoE was found to be expressed by HSPCs, which led to its becoming anchored on the cell surface through interactions with HSPG, similar to how it is known to occur on hepatocytes [14] and macrophages [64], where it participates in cellular lipid transport (Figure 2). ApoE-mediated HSPC proliferative control was shown to be mediated through cellular lipid efflux via ABCA1 and ABCG1, as in their absence, pools of cell surface apoE no longer suppressed HSPC hyperproliferation in mice with dyslipidemia [6]. Although physiological levels of circulating apoE and apoA-I were not found to suppress HSPC proliferation in hyperlipidemic *Ldlr*^−/−^ mice, infusions of larger amounts of recombinant apoA-I containing HDL particles did reduce HSPC proliferation and concomitant monocytosis. The “therapeutic” effect of HDL infusion on suppressing HSPC proliferation was associated with reduced plasma membrane lipid rafts and IL-3/GM-CSF receptor density [6].

## 7. Plasma ApoE and Control of Monocyte Activation in Hyperlipidemic Mice

Human hyperlipidemia often results in apoE accumulation in plasma and atheroma [11,65]. Because of its potent ability to reduce plasma lipid levels in rodents, investigating how elevated apoE levels contribute to regulate atherosclerosis in the setting of hyperlipidemia has been challenging. Work from the authors’ laboratory introduced new strains of mice derived from the HypoE mouse model of conditional *Apoe* expression to address this question [7]. Deleting LDL receptor expression in HypoE mice to generate *Apoe*^h/h^*Ldlr*^−/−^ mice led to spontaneous hyperlipidemia that was similar to levels seen in *Apoe*^−/−^*Ldlr*^−/−^ mice that completely lack apoE. Remarkably, chow-fed *Apoe*^h/h^*Ldlr*^−/−^ mice displayed plasma apoE levels that exceeded those of *Ldlr*^−/−^ mice, suggesting a defect in remnant lipoprotein clearance [7]. Elevated levels of circulating apoE in the hyperlipidemic plasma of *Apoe*^h/h^*Ldlr*^−/−^ mice led to a significant reduction in atherosclerosis in both the aortic root and the abdominal aorta of 5-month-old mice [7]. At that time-point, apoE substantially reduced blood leukocyte counts and led to a two-fold reduction in pro-inflammatory Ly6C^high^ monocytes. ApoE accumulation in hyperlipidemic plasma also led to reduced neutral lipid levels in monocytes, especially among Ly6C^high^ monocytes that displayed reduced levels of adhesion molecules involved in vascular recruitment.

## 8. ApoE Remodels HDL and Improves their Function in Hyperlipidemia

**Figure 3:**
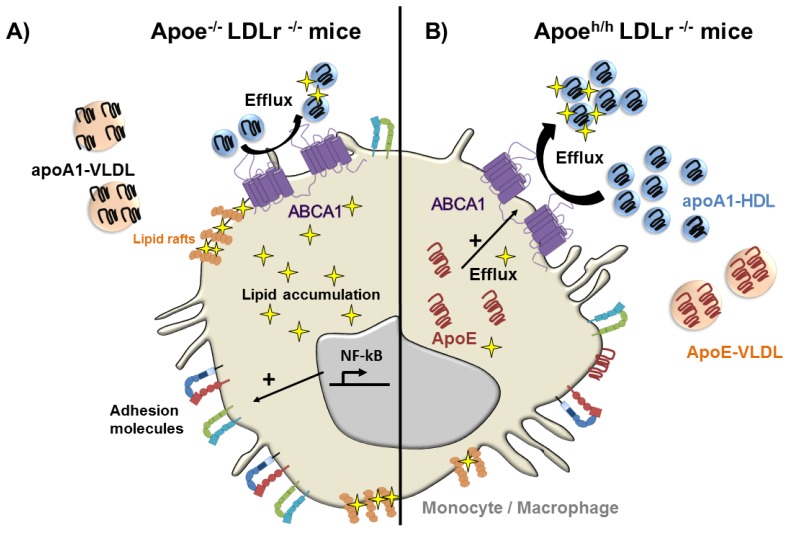
ApoE reduces lipid accumulation and the activation of monocytes and macrophages in hyperlipidemic mice. (**A**) In the absence of apoE, apoA1 distributes to both VLDL and HDL reducing the cholesterol efflux capacity of HDL resulting in cellular lipid accumulation, and thereby NF-κB activation, in monocytes and macrophages; (**B**) Cellular ApoE expression and its accumulation in hyperlipidemic plasma leads to its preferential binding to VLDL causing the displacement of apoA1 that re-distributes to HDL. This increases apoA1-rich HDL in plasma, which more effectively removes cellular lipids from circulating monocytes and macrophages, reducing cellular activation and the expression of cell surface adhesion molecules that contribute to vascular recruitment and atherosclerosis.

The protective effects apoE on reducing blood leukocyte counts, monocyte lipid accumulation and activation in *Apoe*^h/h^*Ldlr*^−/−^ mice was initially linked to its impact on plasma lipoprotein remodeling. In contrast to *Apoe*^−/−^*Ldlr*^−/−^ mouse plasma, where apoA-I distributed among all classes of lipoproteins, in *Apoe*^h/h^*Ldlr*^−/−^, mouse plasma apoE distributed almost exclusively to VLDL, displacing apoA-I and concentrating it on HDL [7]. This resulted in a two-fold increase in HDL-associated cholesterol and apoA-I in *Apoe*^h/h^*Ldlr*^−/−^ mice (Figure 3). Not surprisingly, *Apoe*^h/h^*Ldlr*^−/−^ mouse HDL were found to be more potent at promoting cholesterol efflux form cultured macrophages, and this effect was proposed to protect monocytes from lipid excess. By increasing apoA-I rich HDL in plasma, circulating apoE could also have suppressed HSPC hyperproliferation in *Apoe*^h/h^*Ldlr*^−/−^ mice (Figure 2). Moreover, minor apoE-only HDL such as γLpE could have exerted profound protective effects on reducing cell membrane cholesterol and lipid rafts and thereby growth signaling in HSPCs from these mice. Conversely, the reduced expression levels of the *Apoe* allele in *Apoe*^h/h^*Ldlr*^−/−^ mice likely extended to HSPCs and could explain the more modest reduction in monocytosis observed in this study [7] than in findings reported by Murphy et al. in which HSPC expressed physiological levels of apoE [6]. It is also important to question whether beneficial effects of apoE on suppressing hematopoiesis and monocyte activation derive solely from cellular lipid loss. Hematopoiesis control via apoE could also derive from cellular signaling including through the VLDLR as described by Baitsch et al. [61], or microRNA regulatory pathways, as discussed below.

## 9. ApoE Regulation of Myeloid Cells Signaling via MicroRNA: Impact on Atherosclerosis

**Figure 4:**
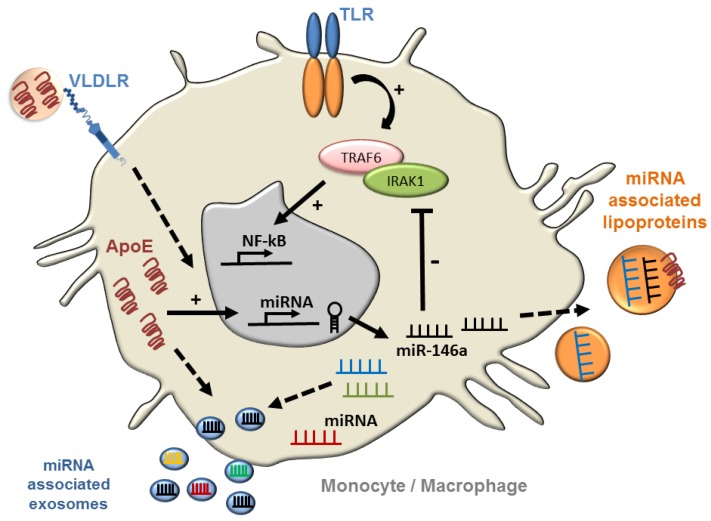
ApoE regulation of NF-κB signaling in monocytes and macrophages via microRNA. ApoE expression has been shown to control myeloid cell activation by increasing cellular miR-146a that suppresses TLR-driven NF-κB signaling by reducing levels of TRAF6 and IRAK1 [10]. Further studies will be required to explore whether lipoprotein associated apoE can contribute to microRNA regulation through receptor-mediated signaling, and whether the source of apoE impacts on the microRNA content of exosomes and plasma lipoproteins to communicate signaling at a distance.

MicroRNAs have emerged as key regulators of inflammation and inflammatory diseases including atherosclerosis [66,67,68]. Studies from the authors’ laboratory [10] have recently uncovered evidence linking cellular apoE expression to enhanced miR-146a levels in macrophages and monocytes that suppress inflammation and atherosclerosis in hyperlipidemic mice (Figure 4). Indeed, among the numerous microRNA that are known to regulate inflammation, miR-146a is established as a critical regulator of myeloid cell activation and expansion [69,70,71]. It also controls the balance between pro- and anti-inflammatory monocytes by down-regulating the expression of the transcription factor RelB that controls the proliferation of Ly-6C^high^ monocytes, which are recognized for their inflammatory [72] and atherogenic properties [73]. MiR-146a is also recognized for its ability to potently suppress acute inflammatory challenges by reducing TLR-driven NF-κB signaling in macrophages and in hematopoietic stem cells [69,71,74]. This function is crucial to prevent an immunological overload and fatal inflammation following a bout of sepsis or LPS injection [70]. The control of miR146a expression by apoE provides new insight to explain the susceptibility of *Apoe*^−/−^ mice to atherosclerosis [75,76] and sepsis [46,77]. Interestingly, the ability of apoE to regulate cellular microRNA levels is not limited to myeloid cells. Studies have shown that apoE can reduce levels of miR-221/222 in smooth muscle cell to suppress their proliferative capacity [8] while increasing levels of miR-145 to reduce aortic stiffening and thereby the recruitment of monocyte-derived macrophages to the vessel wall [9]. Because apoE is known to suppress endothelial cell activation [34], it is possible that it could be doing so in part by raising cellular miR-146a levels that can suppress endothelial dysfunction [78]. Such enrichment of miR146a or even apoE itself in endothelial cells could derive from its delivery via exosomes secreted by macrophages (Figure 4). Support for such concepts comes from the results of a study that recently reported the delivery of macrophage-apoE to tumor cells via exosomes that controlled migratory phenotypes [79].

Collectively, findings of apoE-regulation of microRNA signaling provide new mechanistic properties to explain its capacity to suppresses atherosclerosis beyond reducing plasma lipid levels or enhancing cellular lipid efflux. In fact, this new mode of cellular regulation by apoE could explain results of earlier reports that documented its capacity to suppress type I inflammation [46] and promote atherosclerosis regression beyond reducing plasma lipid levels [19]. Additionally, because apoE participates in plasma lipoprotein remodeling [7], it could impact their microRNA repertoire and ultimately their delivery to target cells (Figure 4), a process that has recently been shown to suppress vascular inflammation in mice [80]. Preliminary data from the authors’ laboratory support this possibility that could in part explain the observed reduced endothelial activation and atherosclerosis in hyperlipidemic mice that accumulate apoE in plasma [7]. Together, such findings highlight the complexity through which apoE contributes to regulate microRNA signaling to suppress inflammation and atherosclerosis in the absence of plasma lipid reduction. Future studies will be required to address the relevance of this and other protective mechanisms through which apoE can control atherosclerosis in humans including in an isoform-specific manner.

## 10. Conclusions

More than 40 years after its discovery, apoE remains actively studied owing to its remarkable number of emerging pleiotropic properties, many of which contribute to the suppression of inflammation and atherosclerosis. Far beyond its well-known ability to clear cholesterol-rich lipoproteins from plasma or enhance the release of cellular lipid from macrophage foam cells, apoE is increasingly being recognized for its ability to control cellular signaling in cells of the immune system and vessel wall. Our understanding of the vast cellular regulatory networks controlled by apoE is just beginning to emerge. Past and more recent evidence demonstrates that apoE can impact numerous forms of cellular signaling that now include microRNA controlled cellular gene expression, including in monocytes, macrophages and smooth muscle cells. Therefore, monitoring apoE-relevant microRNA signatures may provide new biomarkers for early atherosclerosis detection. Furthermore, the use of mimics and antagonists of apoE-relevant microRNA delivered via nanoparticles including liposomes and exosomes, could offer new therapeutic approaches to control atherosclerosis cardiovascular diseases.
